# Exponential-modified discrete Lindley distribution

**DOI:** 10.1186/s40064-016-3302-2

**Published:** 2016-09-26

**Authors:** Mehmet Yilmaz, Monireh Hameldarbandi, Sibel Acik Kemaloglu

**Affiliations:** 1 Department of Statistics, Faculty of Science, Ankara University, 06100 Tandogan, Ankara Turkey; 2 Graduate School of Natural and Applied Sciences, Ankara University, 06110 Diskapi, Ankara Turkey

**Keywords:** Modified discrete Lindley distribution, Exponential- modified, Discrete Lindley distribution, Method of moments, Maximum likelihood estimation, EM-algorithm, 46N30, 62F10, 60E05

## Abstract

In this study, we have considered a series system composed of stochastically independent M-component where M is a random variable having the zero truncated modified discrete Lindley distribution. This distribution is newly introduced by transforming on original parameter. The properties of the distribution of the lifetime of above system have been examined under the given circumstances and also parameters of this new lifetime distribution are estimated by using moments, maximum likelihood and EM-algorithm.

## Background

Under the name of the “*new lifetime distribution*”, about 400 studies have been done in the recent 5 years. In particular, the compound distributions obtained by exponential distribution are applicable in the fields such as electronics, geology, medicine, biology and actuarial. Some of these works can be summarized as follows: Adamidis and Loukas (1998) and Adamidis et al. (2005) introduced a two-parameter lifetime distribution with decreasing failure rate by compounding exponential and geometric distribution. In the same way, exponential-Poisson (EP) and exponential-logarithmic (EL) distributions were given by Kus (2007) and Tahmasbi and Rezaei (2008), respectively. Chahkandi and Ganjali (2009) introduced exponential-power series distributions (EPS). Barreto-Souza and Bakouch (2013) introduced a new three-parameter distribution by compounding exponential and Poisson–Lindley distributions, named the exponential Poisson–Lindley (EPL) distribution. Exponential-Negative Binomial distribution is introduced by Hajebi et al. (2013). Furthermore, Gui et al. (2014) have considered the Lindley distribution which can be described as a mixture of the exponential and gamma distributions. This idea has helped them to propose a new distribution named as Lindley–Poisson by compounding the Lindley and Poisson distributions.

Because most of those distributions have decreasing failure rate. They have important place in reliability theory. Lots of those lifetime data can be modelled by compound distributions. Although these compound distributions are quite complex, new distributions can fit better than the known distributions for modelling lifetime data.

Probability mass function of the discrete Lindley distribution obtained by discretizing the continuous survival function of the Lindley distribution (Gómez-Déniz and Calderín-Ojeda 2011; Eq. 3, Bakouch et al. 2014; Eq. 3). This discrete distribution provided by authors above, is quite a complex structure in terms of parameter. In order to overcome problems in estimation process of the parameter of Lindley distribution, we propose a modified discrete Lindley distribution. Thus, estimation process of the parameters using especially the EM algorithm was facilitated. Afterwards, we propose a new lifetime distribution with decreasing hazard rate by compounding exponential and modified-zero-truncated discrete Lindley distributions.

This paper is organized as follows: In “Construction of the model” section, we propose the two-parameter exponential-modified discrete Lindley (EMDL) distribution, by mixing exponential and zero truncated modified discrete Lindley distribution, which exhibits the decreasing failure rate (DFR) property. In “Properties of EMDL distribution” section, we obtain moment generating function, quantile, failure rate, survival and mean residual lifetime functions of the EMDL. In “Inference” section, the estimation of parameters is studied by some methods such as moments, maximum likelihood and EM algorithm. Furthermore, information matrix and observed information matrix are also discussed in this section. The end of this section includes a detailed simulation study to see the performance of Moments (with lower and upper bound approximations), ML and EM estimates. Illustrative examples based on three real data sets are provided in “Applications” section.

## Construction of the model

In this section, we first give the definition of the discrete Lindley distribution introduced by Gómez-Déniz and Calderín-Ojeda (2011) and Bakouch et al. (2014). We have achieved a more simplified discrete distribution than discrete Lindley distribution by taking $1-\theta$ instead of $e^{-\theta }$ in subsequent definition. Thus, we introduce a new lifetime distribution by compounding Exponential and Modified Discrete Lindley distributions, named the Exponential-Modified Discrete Lindley (EMDL) distribution.

### Discrete Lindley distribution

A discrete random variable *M* is said to have Lindley distribution with the parameter $\theta >0$, if its probability mass function (p.m.f) is given by

1 $$P\left( M=m\right) =\frac{e^{-m\theta }}{1+\theta }\left( \theta \left( 1-2e^{-\theta }\right) +\left( 1-e^{-\theta }\right) \left( 1+\theta m\right) \right) , \quad m=0,1,2,\ldots$$

The cumulative distribution function of *M* will be given by

$$P\left( M\le m\right) =1-\frac{1+2\theta +\theta m}{1+\theta }e^{-\left( m+1\right) \theta },\ \ \ \ \ \ m=0,1,2,\ldots$$

### Modified discrete Lindley distribution

If $\theta$ is limited to the range (0, 1), then we replace $exp(-\theta )$ by $1-\theta$ using the first degree Taylor expansion of $exp(-\theta )$ in (1). The new discrete distribution is specified by the following probability mass function:

2 $$P\left( M=m\right) =\frac{{\theta }^2}{1+\theta }{\left( 1-\theta \right) }^m\left( m+2\right),$$

for $0<\theta <1$ and $m=0,1,2,\ldots$. We call this distribution Modified Discrete Lindley (MDL).

#### **Theorem 1**

*MDL distribution can be represented as a mixture of geometric and negative binomial distributions with mixing proportion is*$\frac{\theta }{1+\theta }$*, and a common success rate*$\theta$.

#### *Proof*

**Fig. 1:**
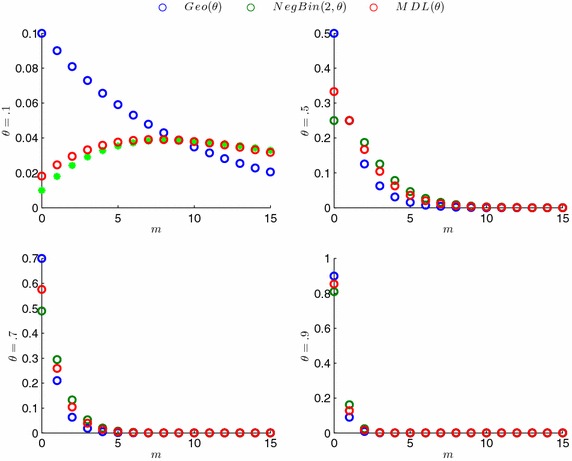
P.m.f of geometric, negative binomial and modified discrete Lindley

If p.m.f in (2) is rewritten as the following form

$$P\left( M=m\right) =\frac{\theta }{1+\theta }\left[ \theta {\left( 1-\theta \right) }^m\right] +\frac{1}{1+\theta }\left[ {\theta }^2\left( m+1\right) {\left( 1-\theta \right) }^m\right] =w_1f_1\left( m\right) +w_2f_2\left( m\right),$$

then $f_1$ indicates p.m.f of a geometric random variable with success probability $\theta$ and $f_2$ indicates p.m.f of a negative binomial random variable which denotes the number of trials until the second success, with common success probability $\theta$. $w_1=\frac{\theta }{1+\theta }$ and $w_2=\frac{1}{1+\theta }$ denote component probabilities; in other words these are called the mixture weights (Fig. 1). $\square$

Note that MDL distribution has an increasing hazard rate while a geometric distribution has a constant hazard rate. So, MDL distribution is more useful than geometric distribution for modelling the number of rare events.

When the $\theta$ is closed to zero, then MDL can occure different shapes than the p.m.f of a Geometric distribution. This situation made the distribution thinner right tail than a distribution which is compounded with exponential distribution. Thus, this proposed compound distribution can be usefull for modelling lifetime data such as time interval between successive earthquakes, time period of bacteria spreading, recovery period of the certain disease.

### Exponential modified discrete Lindley distribution

Suppose that *M* is a zero truncated *MDL* random variable with probablity mass function $$\pi \left( m\right) =P(M=m$$$$\vert {M>0})=\frac{{\theta }^2}{\left( 1+2\theta \right) }{\left( 1-\theta \right) }^{m-1}\left( m+2\right)$$ and $$X_1,X_2,\ldots, X_M$$ are i.i.d. with probability density function $$h\left( x;\beta \right) =\beta e^{-\beta x},\ x>0$$. Let $$X=min\left( X_1,X_2,\ldots ,X_M\right)$$, then $$g\left( x\vert {m};\beta \right) =m\beta e^{-m\beta x}$$ and $$g\left( x,m\right) =g\left( x\vert {m}\right) \pi (m)=\frac{\beta {\theta }^2}{\left( 1+2\theta \right) }m\left( m+2\right) {\left( 1-\theta \right) }^{m-1}e^{-m{\beta}x }$$.

Thus, we can obtain the marginal probability density function of *X* as

3 $$f\left( x;\theta ,\beta \right) =\frac{{\theta }^2}{1+2\theta} \frac{\beta e^{-\beta x}\left( 3-\left( 1-\theta \right) e^{-\beta x}\right) }{{\left( 1-\left( 1-\theta \right) e^{-\beta x }\right)}^3},\quad x>0$$

where *θ* ∈ (0, 1) and *β* > 0. Henceforth, the distribution of the random variable *X* having the p.d.f in (3) is called shortly EMDL. By changing of variables $r=\left( 1-\theta \right) e^{-\beta x}$ in cumulative integration of (3), the distribution function can be found as follows:

$$F\left( x;\theta ,\beta \right) =1-\left[ \frac{{\theta }^2}{1+2\theta }\frac{e^{-\beta x}\left( 3-2\left( 1-\theta \right) e^{-\beta x}\right) }{{\left( 1-\left( 1-\theta \right) e^{-\beta x}\right) }^2}\right].$$

**Fig. 2:**
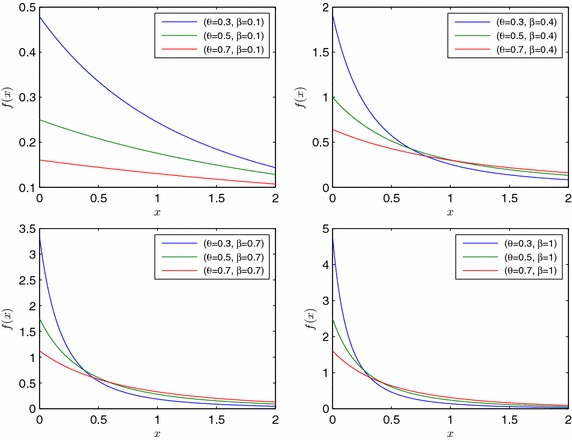
P.d.f of *EMDL* random variable for different parameter values

Following figure shows different shapes of p.d.f of *EMDL* random variable for various values of $\theta$ and $\beta$ (Fig. 2).

## Properties of *EMDL* distribution

In this section the important characteristics and features in mathematical statistics and realibility which are moment generating function and moments, quantiles, survival, hazard rate and mean residual life functions of the *EMDL* distribution are introduced. We will also give a relationship with Lomax and Exponential-Poisson distributions.

### Moment generating function and moments

Moment generating function of *X* is given by

$$M\left( t\right) =E\left( e^{tx}\right) =\frac{{\theta }^2}{1+2\theta }\sum _{j=1}^{\infty} j\left( j+2\right) {\left( 1-\theta \right) }^{j-1}\frac{\beta }{\beta j-t}$$

for $t<\beta$. Hence a closed form of *k*.th raw moment of *X* is expressed by

$$E\left( X^k\right) =\frac{\Gamma \left( k+1\right) {\theta }^2}{{\beta }^k\left( 1+2\theta \right) }\sum _{j=1}^{\infty }\frac{\left( j+2\right) }{j^k}{\left( 1-\theta \right) }^{j-1},$$

for $k=1,2,\ldots$. Here for $k>1$ raw moments can be calculated numerically for given values of $\theta$ since infinite series above can be represented by *polylog* functions.

First and second raw moments are evaluated respectively as

4 $$E\left( X\right)= \frac{\theta }{\beta \left( 1+2\theta \right) }\left[ 1-\frac{2\theta \ln {\theta }}{1-\theta} \right],$$

5 $$E \left( X^2\right)=\frac{2{\theta }^2}{{\beta }^2\left( 1+2\theta \right) \left( 1-\theta \right) }\left[ -\ln {\theta }+2\sum _{k=1}^{\infty }\frac{{\left( 1-\theta \right) }^k}{k^2}\right]$$

### Quantile function

Quantile function of *X* is obtained simply by inverting $F(x;\theta ,\beta )=q$ as follows

$$x_q=\frac{log\left( 1-\theta \right) -log\left[ \frac{\left( 2\left( 1-q\right) +3A\left( \theta \right) \right) \,-\,\sqrt{9{A\left( \theta \right) }^2+4\left( 1-q\right) A\left( \theta \right) }}{2\left( 1-q\right) +4A\left( \theta \right) }\right] }\beta$$

where $0<q<1$ and $A(\theta )=\frac{{\theta }^2}{(1+2\theta )(1-\theta )}$. In particular, the first quartile of *X* is

$$x_{0.25}=\frac{log\left( 1-\theta \right) -log\left[ \frac{\left( \frac{3}{2}+3A\left( \theta \right) \right)\, -\,\sqrt{9{A\left( \theta \right) }^2+3A\left( \theta \right) }}{\frac{3}{2}+4A\left( \theta \right) }\right] }{\beta },$$

the median of *X* is

$$x_{0.5}=\frac{log\left( 1-\theta \right) -log\left[ \frac{\left( 1+3A\left( \theta \right) \right) \,-\,\sqrt{9{A\left( \theta \right) }^2+2A\left( \theta \right) }}{1+4A\left( \theta \right) }\right] }{\beta },$$

and the third quartile of *X* is

$$x_{0.75}=\frac{log\left( 1-\theta \right) -log\left[ \frac{\left( \frac{1}{2}+3A\left( \theta \right) \right) \,-\,\sqrt{9{A\left( \theta \right) }^2+A\left( \theta \right) }}{\frac{1}{2}+4A\left( \theta \right) }\right] }{\beta }.$$

### Survival, hazard rate and mean residual life functions

**Fig. 3:**
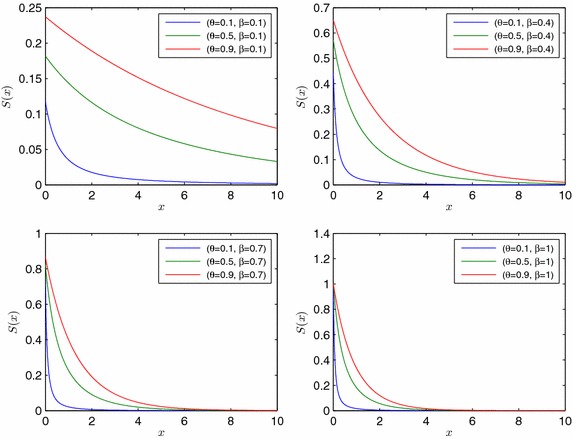
Survival function of *EMDL* random variable for selected parameter values

The survival function of *X* is given by (Fig. 3)

6 $$S\left( x\right) =\frac{{\theta }^2}{1+2\theta }\left( \frac{e^{-\beta x}\left( 3-2\left( 1-\theta \right) e^{-\beta x}\right) }{{\left( 1-\left( 1-\theta \right) e^{-\beta x}\right) }^2}\right).$$

**Fig. 4:**
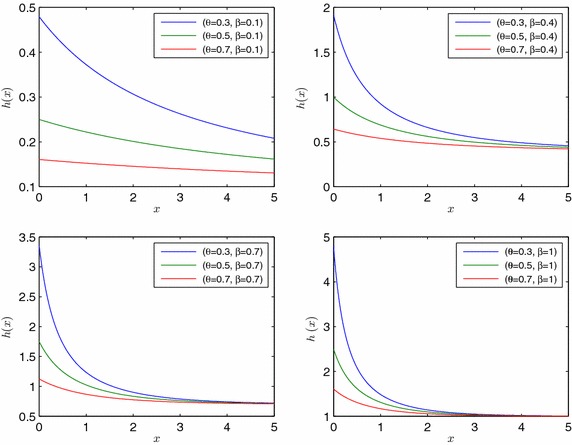
Hazard rate function of EMDL random variable for selected parameter values

From (3) and (6) it is easy to verify that the hazard rate function of *X* is

7 $$\begin{aligned} h\left( x\right)&=\,\frac{f\left( x\right) }{S\left( x\right) } =\beta \frac{\left( 3-r\right) }{\left( 1-r\right) \left( 3-2r\right) }=\beta \left[ 1+2\frac{r\left( 2-r\right) }{\left( 1-r\right) \left( 3-2r\right) }\right] \\&=\,\beta \left[ \frac{2}{\left( 1-r\right) }-\frac{3}{\left( 3-2r\right) }\right] \end{aligned}$$

with $h\left( 0\right) =\frac{\beta \left( 2+\theta \right) }{\theta \left( 1+2\theta \right) }\ge \beta$ and $\lim _{x\rightarrow \infty }{h\left( x\right) }=\beta$ where $r=\left( 1-\theta \right) e^{-\beta x}$. As it can be seen immediately from last two statements on the right side of (7), *h*(*x*) is a monotonically decreasing function and bounded from below with $\beta$ (see Fig. 4).

The mean residual life function of *X* is given by

$$\begin{aligned} mrl\left( x\right)&=E\left( X-x\vert {X>x}\right) \ \\&=\frac{1}{\beta }\ \frac{r\left( 1-r\right) -2{\left( 1-r\right) }^2ln\left( 1-r\right) }{3r-2r^2} \end{aligned}$$

where $r=\left( 1-\theta \right) e^{-\beta x}$. Note that $mrl\left( x\right) \le \frac{1}{\beta }$ holds for $x>0$. We can see this result immediately below by letting $-ln\left( 1-r\right) =\int _{1-r}^1\frac{1}{z}dz$. Then applying the mean value theorem, we have the upper bound for $-ln\left( 1-r\right)$ as $\frac{r}{1-r}$. If this upper bound is written above, then

$$mrl\left( x\right) \le \frac{1}{\beta }\ \frac{3\left( 1-r\right) }{3-2r}\le \frac{1}{\beta }.$$

**Fig. 5:**
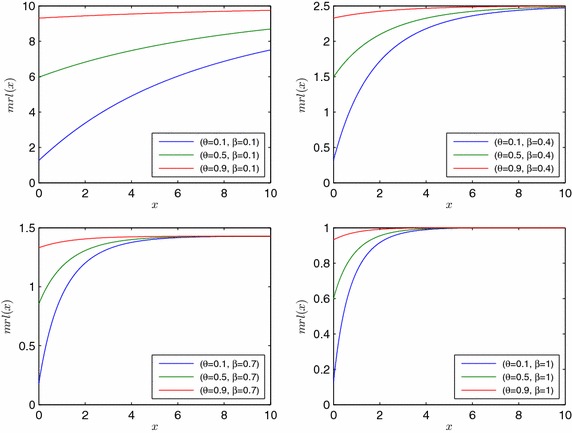
Mrl function of EMDL random variable for different parameter values

We have the following graphs of *mrl*(*x*) for different values of parameter $\theta$ and $\beta$ (Fig. 5).

### Relationship of the other distribution 

Let consider the following transformation of *X*

$$Y=\frac{e^{\beta X}-1}{1-\theta }.$$

Then the probability density function of *Y* can be obtained as

$$f_Y\left( y\right) =\frac{3\theta }{1+2\theta }\left( \frac{\frac{\theta }{1-\theta }}{{\left( y+\frac{\theta }{1-\theta }\right) }^2}\right) +\frac{1-\theta }{1+2\theta }\left( \frac{2{\left( \frac{\theta }{1-\theta }\right) }^2}{{\left( y+\frac{\theta }{1-\theta }\right) }^3}\right)$$

It can be easily seen that distribution of *Y* is a mixture of two Lomax distributions with common scale paramater $\frac{\theta }{1\,-\,\theta }$, and $\alpha =1$ and $\alpha =2$ respectively. Thus, $\frac{3\theta }{1\,+\,2\theta }$ and $\frac{1-\theta }{1+2\theta }$ represent the weight probabilities of mixture components.

## Inference

In this section the estimation techniques of the parameters of the *EMDL* distribution are studied using the moments, maximum likelihood and EM algorithm. In particular, because first two moments of the distribution have a very complex structure, we have developed bounds to get a solution more easily. Fisher information matrix and asymptotic confidence ellipsoid for the parameters $\theta$ and $\beta$ are also obtained. A detailed simulation study based on four estimation mehods is located at the end of this section.

### Estimation by moments

Let $X_1,\ X_2,\dots ,{X}_n$ be a random sample from *EMDL* distribution and $m_1$ and $m_2$ represent the first two sample moments. Then from (4) and (5), we will have the following system of equations

8 $$m_1= \frac{\theta }{\beta \left( 1+2\theta \right) }\left[ 1-\frac{2\theta \ln {\theta }}{1-\theta }\right] ,$$

9 $$m_2= \frac{2{\theta }^2}{{\beta }^2\left( 1+2\theta \right) \left( 1-\theta \right) }\left[ -\ln {\theta }+2\ I\left( \theta \right) \right] .$$

where $$I\left( \theta \right) =\sum _{k=1}^{\infty }\frac{{\left( 1-\theta \right) }^k}{k^2}$$.

Moment estimates of $\theta$ and $\beta$ can be obtained by solving equations above. However, Eqs. (8) and (9) have no explicit analytical solutions for the parameters. Thus, the estimates can be obtained by means of numerical procedures such as Newton-Raphson method. Since we can only get the symbolic computation for $I\left( \theta \right)$, the calculation process takes too long during simulations. Therefore, we will find the lower and upper bounds for $I\left( \theta \right)$.

#### **Theorem 2**

*For*$\theta \in \left[ 0,1\right]$, $I\left( \theta \right)$*lies between*$\frac{\theta }{1-\theta }\ln {\left( \theta \right) }+\frac{3-\theta }{2}$*and*$\frac{\theta \left( 2-\theta \right) }{2\left( 1-\theta \right) }\ln {\left( \theta \right) }+\frac{7-5\theta }{4}$*i.e.*

$$\frac{\theta }{1-\theta }\ln {\left( \theta \right) }+\frac{3-\theta }{2}\le I\left( \theta \right) \le \frac{\theta \left( 2-\theta \right) }{2\left( 1-\theta \right) }\ln {\left( \theta \right) }+\frac{7-5\theta }{4}$$

#### *Proof*

(lower bound) Let write inequality $k^2\le k(k+1)$ for all *k*, then $\frac{{\left( 1-\theta \right) }^k}{k^2}\ge \frac{{\left( 1-\theta \right) }^k}{k(k+1)}$ holds. We have the following lower bound for $I\left( \theta \right)$ when summation is made over *k*

$$I\left( \theta \right) \ge \sum _{k=1}^{\infty }\frac{{\left( 1-\theta \right) }^k}{k\left( k+1\right) }=\sum _{k=1}^{\infty }\frac{{\left( 1-\theta \right) }^k}{k}-\sum _{k=1}^{\infty }\frac{{\left( 1-\theta \right) }^k}{k+1}.$$

According to convergence test (comparison test) of infinite series, since $\sum _{k=1}^{\infty }{\left( 1-\theta \right) }^k$ is a convergent geometric series, two infinite series in the right hand side of inequality above are both convergent. By using Fubini’s theorem for these series respectively we have

10 $$\begin{aligned} \sum _{k=2}^{\infty }\frac{{\left( 1-\theta \right) }^k}{k}&=\sum _{k=2}^{\infty }\left( \int _{\theta }^1{\left( 1-z\right) }^{k-1}dz\right) =\int _{\theta }^1\left( \sum _{k=2}^{\infty }{\left( 1-z\right) }^{k-1}\right) dz \\&=\theta -\ln {\left( \theta \right) }-1\ \end{aligned}$$

and

11 $$\begin{aligned} \sum _{k=2}^{\infty }\frac{{\left( 1-\theta \right) }^k}{k+1}&=\frac{1}{\left( 1-\theta \right) }\sum _{k=2}^{\infty }\left( \int _{\theta }^1{\left( 1-z\right) }^kdz\right) =\frac{1}{\left( 1-\theta \right) }\int _{\theta }^1\left( \sum _{k=2}^{\infty }{\left( 1-z\right) }^k\right) dz \\&=\frac{-2\ln {\left( \theta \right) }+4\theta -{\theta }^2-3}{2\left( 1-\theta \right) } \end{aligned}$$

By subtracting first term from the second and adding $\left( 1-\theta \right)$, then we get the lower bound for $I\left( \theta \right)$.

(upper bound) Let write inequality $k^2\ge k^2-1$ for all $k=2,3,\ldots$, then we have the upper bound for $\sum _{k=2}^{\infty }\frac{{\left( 1-\theta \right) }^k}{k^2}$ as below:

12 $$\sum _{k=2}^{\infty }\frac{{\left( 1-\theta \right) }^k}{k^2}\le \frac{1}{2}\left[ \sum _{k=2}^{\infty }\frac{{\left( 1-\theta \right) }^k}{k-1}-\sum _{k=2}^{\infty }\frac{{\left( 1-\theta \right) }^k}{k+1}\right]$$

Let’s add and subtract the term $\sum _{k=2}^{\infty }\frac{{\left( 1-\theta \right) }^k}{k}$ in bounds above, then

$$\left( \sum _{k=2}^{\infty }\frac{{\left( 1-\theta \right) }^k}{k-1}-\sum _{k=2}^{\infty }\frac{{\left( 1-\theta \right) }^k}{k}\right) +\left( \sum _{k=2}^{\infty }\frac{{\left( 1-\theta \right) }^k}{k}-\sum _{k=2}^{\infty }\frac{{\left( 1-\theta \right) }^k}{k+1}\right).$$

First term can be rewritten following form

$$\begin{aligned} \sum _{k=2}^{\infty }\frac{{\left( 1-\theta \right) }^k}{k-1}-\sum _{k=2}^{\infty }\frac{{\left( 1-\theta \right) }^k}{k}&={\left( 1-\theta \right) }^2+\sum _{k=3}^{\infty }\frac{{\left( 1-\theta \right) }^k}{k-1}-\frac{{\left( 1-\theta \right) }^2}{2}-\sum _{k=3}^{\infty }\frac{{\left( 1-\theta \right) }^k}{k}\\&=\frac{{\left( 1-\theta \right) }^2}{2}+\left( 1-\theta \right) \left( \sum _{k=2}^{\infty }\frac{{\left( 1-\theta \right) }^k}{k}-\sum _{k=2}^{\infty }\frac{{\left( 1-\theta \right) }^k}{k+1}\right) \end{aligned}$$

Thus, (12) can be expressed by

$$\frac{{\left( 1-\theta \right) }^2}{2}+\left( 2-\theta \right) \left( \sum _{k=2}^{\infty }\frac{{\left( 1-\theta \right) }^k}{k}-\sum _{k=2}^{\infty }\frac{{\left( 1-\theta \right) }^k}{k+1}\right) .$$

The latter is combined with the expressions (10) and (11) together then we have

$$\frac{{\left( 1-\theta \right) }^2}{2}+\left( 2-\theta \right) \left( \frac{\theta }{1-\theta }\ln {\left( \theta \right) }+\frac{1+\theta }{2}\right).$$

If this result is placed in position in the brackets in the expression (12), and adding the term $\left( 1-\theta \right)$, then the upper bound is obtained.

**Fig. 6:**
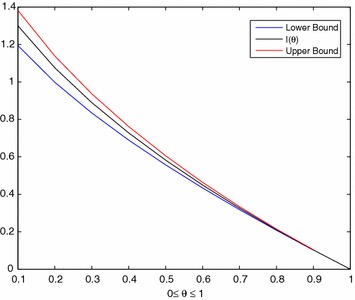
Lower and upper bounds for $I(\theta )$

Graph below shows that these bounds are eligible for $I\left( \theta \right)$, so, this leads us to solve moment estimate by using these bounds (Fig. 6).

Now let’s go back to the moments estimation problem. From the Eq. (8) we get the equality for $\beta$ and replace it in (9), then we have the following equation to get a solution for $\theta$

$$\left( 1+2\theta \right) \left( 1-\theta \right) \frac{-\ln {\left( \theta \right) }+2I\left( \theta \right) }{{\left( 1-\theta -2\theta \ln {\left( \theta \right) }\right) }^2}-\frac{m_2}{2m_1}=0.$$

Solution was obtained by putting lower and upper limits in place of $I\left( \theta \right)$, and applying Newton Raphson’s method.

### Estimation by maximum likelihood 

Let $x\ =\ (x_1,\ x_2,\ldots , x_n)$ be an observation of size *n* from the *EMDL* distribution with parameters $\theta$ and $\beta$. The log likelihood $\ell$ = $\ell (\theta ,\beta ;\ x)$ for $(\theta ,\beta )$ is

13 $$\begin{aligned} \ell \left( \theta ,\beta ;x\right)&=n\ln {\beta }+n\ln {\left( \frac{{\theta }^2}{1+2\theta }\right) }-\beta \sum _{i=1}^nx_i+\sum _{i=1}^n\ln {\ \left( 3-\left( 1-\theta \right) e^{-\beta x_i}\right) } \\&\quad -3\sum _{i=1}^n\ln {\ \left( 1-\left( 1-\theta \right) e^{-\beta x_i}\right) } \end{aligned}$$

and subsequently differentiating (13) with respect to $\theta$ and $\beta$ yields the likelihood equations for $(\theta ,\beta )$

$$\begin{aligned} \frac{\partial \ell }{\partial \theta }= & \frac{2n\left( 1+\theta \right) }{\theta \left( 1+2\theta \right) }+\sum _{i=1}^n\frac{e^{-\beta x_i}}{3-\left( 1-\theta \right) e^{-\beta x_i}}-3\sum _{i=1}^n\frac{e^{-\beta x_i}}{1-\left( 1-\theta \right) e^{-\beta x_i}}=0 \\ \frac{\partial \ell }{\partial \beta }= & \frac{n}{\beta }-\sum _{i=1}^nx_i+\sum _{i=1}^n\frac{x_i(1-\theta )e^{-\beta x_i}}{3-\left( 1-\theta \right) e^{-\beta x_i}}-3\sum _{i=1}^n\frac{x_i(1-\theta )e^{-\beta x_i}}{1-\left( 1-\theta \right) e^{-\beta x_i}}=0 \end{aligned}$$

The solution of two equations above does not have a closed form, therefore numerical techniques can be used to solve the above system of equations.

We investigate below conditions for the solution of this system of equations for $\beta$ and $\theta$.

#### **Proposition 1**

*If*$\frac{n}{2}<\sum _{i=1}^{n}e^{-\beta x_{i}}$*, then the equation*$\partial \ell /\partial \theta =0$*has at least one root in*$\left( 0,1\right)$*, where*$\beta$*is the true value of the parameter.*

#### *Proof*

Let $\omega \left( \theta \right)$ denote the function on the RHS of the expression $\partial \ell /\partial \theta$, then it is clear that $\lim \limits _{\theta \rightarrow 0}\omega \left( \theta \right) =+\infty$ and $\lim \limits _{\theta \rightarrow 1}\omega \left( \theta \right) =\frac{4n }{3}+\frac{1}{3}\sum _{i=1}^{n}e^{-\beta x_{i}}-3\sum _{i=1}^{n}e^{-\beta x_{i}}$. Therefore, the equation $\omega \left( \theta \right) =0$ has at least one root in $\left( 0,1\right)$, if $\frac{n}{2}-\sum _{i=1}^{n}e^{-\beta x_{i}}<0$. $\square$

#### **Proposition 2**

*If*$\theta$*is the true value of the parameter, the root of the equation*$\partial \ell /\partial \beta =0$*lies in the interval*$\left[ \frac{1}{ \overline{X}}\frac{\theta }{\left( 3-2\theta \right) },~\frac{1}{\overline{X} }\frac{2+\theta }{\left( 1+2\theta \right) }\right]$.

#### *Proof*

Let $\omega \left( \beta \right)$ denote the function on the RHS of the expression $\partial \ell /\partial \beta$, then

$$\begin{aligned} \omega \left( \beta \right) \le \frac{n}{\beta }-\sum _{i=1}^{n}x_{i}+\sum _{i=1}^{n}\frac{x_{i}(1-\theta )e^{-\beta x_{i}}}{3-(1-\theta )e^{-\beta x_{i}}} \end{aligned}$$

Note that $3-(1-\theta )e^{-\beta x_{i}}\ge 2+\theta$ and $e^{-\beta x_{i}}\le 1$. Hence,

$$\begin{aligned} \omega \left( \beta \right) \le \frac{n}{\beta }-\sum _{i=1}^{n}x_{i}+\frac{(1-\theta )}{2+\theta }\sum _{i=1}^{n}x_{i}. \end{aligned}$$

Therefore, $\omega \left( \beta \right) \le 0$ when $\beta \ge \frac{1}{ \overline{x}}\frac{2+\theta }{1+2\theta }$. On the other hand,

$$\begin{aligned} \omega \left( \beta \right) \ge \frac{n}{\beta }-\sum _{i=1}^{n}x_{i}-3\sum _{i=1}^{n}\frac{x_{i}(1-\theta )e^{-\beta x_{i}}}{1-(1-\theta )e^{-\beta x_{i}}}. \end{aligned}$$

By noting $1-(1-\theta )e^{-\beta x_{i}}\ge \theta$ and $e^{-\beta x_{i}}\le 1$. Hence,

$$\begin{aligned} \omega \left( \beta \right) \ge \frac{n}{\beta }-\left( 1+\frac{3\left( 1-\theta \right) }{\theta }\right) \sum _{i=1}^{n}x_{i}. \end{aligned}$$

Therefore, $\omega \left( \beta \right) \ge 0$ when $\beta \le \frac{1}{ \overline{x}}\frac{\theta }{3-2\theta }$. Thus, there is at least one root of $\omega \left( \beta \right) =0$ in the interval $\left( \frac{1}{ \overline{X}}\frac{\theta }{\left( 3-2\theta \right) },~\frac{1}{\overline{X} }\frac{2+\theta }{\left( 1+2\theta \right) }\right)$. Recently, EM algorithm has been used by several authors to find the ML estimates of compound distributions’ parameters. EM algorithm which is used to make maximizing the complete data loglikelihood is useful when observed log likelihood equations are difficult to solve. However EM algorithm plays a crucial role for getting parameter estimates in such compound distribution as long as equations obtained in E-step are more simple and clear. $\square$

### Estimation by EM algorithm

The hypothetical complete-data (*x*, *m*) density function is given by

$$\begin{aligned} f\left( x,m;\theta ,\beta \right) =\frac{\beta {\theta }^2}{1+2\theta }m\left( m+2\right) {\left( 1-\theta \right) }^{m-1}e^{-\beta mx} \end{aligned}$$

for $x\epsilon R_+, m=1,2,\ldots ,\ \theta \epsilon \left( 0,1\right) , \beta >0$. Here, $\theta$ and $\beta$ are the parameters of the exponential-zero truncated Lindley distribution. According to E-step of EM cycle, we need to compute the conditional expectation of *M* with given $X=x$. Therefore, immediately let’s write conditional probability mass function as below:

14 $$\begin{aligned} P\left( M=m \vert {x};\theta ,\beta \right) =\frac{{\left( 1-r\right) }^3}{3-r}m\left( m+2\right) r^{m-1},\ \end{aligned}$$

for $m=1,2,\ldots$, where $r=\left( 1-\theta \right) e^{-\beta x}$. By using equation (14), we can find the conditional expectation of *M* to complete E-step as

$$\begin{aligned} \delta (x;\theta ,\beta )=E\left( M\vert {x};\theta ,\beta \right) =6\left[ \frac{1}{\left( 3-r\right) \left( 1-r\right) }\right] -1.\ \end{aligned}$$

M-step of each iteration requires maximization of complete-data likelihood function defined over $\left( \theta ,\beta \right)$. Let’s $\ell _c$ indicate complete-data log likelihood function, i.e. $\ln {L} \left( \theta ,\beta ; x, m\right)$ then

$$\begin{aligned} \ell _c&=n\ln {\beta }+2n\ln {\theta }-n\ln {\left( 1+2\theta \right) }+\sum _{i=1}^n\ln {\left( m_i\left( m_i+2\right) \right) }\\&\quad +\ln {\left( 1-\theta \right) }\sum _{i=1}^n\left( m_i-1\right) -\beta \sum _{i=1}^nm_ix_i.\ \end{aligned}$$

Hence, the likelihood equations can be verified by evaluating $\frac{\partial \ell _c}{\partial \theta }=0$ and $\frac{\partial \ell _c}{\partial \beta }=0$ as below:

15 $$\begin{aligned} &\frac{2\left( 1-\theta \right) }{\theta }-\frac{2\left( 1-\theta \right) }{1+2\theta }+1=\frac{\sum _{i=1}^nm_i}{n} \\ &\frac{1}{\beta }=\frac{\sum _{i=1}^nm_ix_i }{n} \end{aligned}$$

The M-step is completed with the missing observations of $M_i$ replaced by $\delta (x_i;{\theta }^{\left( t\right) },{\beta }^{\left( t\right) }\ )$. Thus, iterative solution of the system of equations in (15) is given by

$$\begin{aligned}&{\theta }^{\left( t+1\right) } =\frac{\left( 1-\frac{\sum _{i=1}^n{k_i}^{\left( t\right) }}{n}\right) +\sqrt{{\left( 1-\frac{\sum _{i=1}^n{k_i}^{\left( t\right) }}{n}\right) }^2+16\frac{\sum _{i=1}^n{k_i}^{\left( t\right) }}{n}}}{4\frac{\sum _{i=1}^n{k_i}^{\left( t\right) }}{n}}\\&{\beta }^{\left( t+1\right) }=\frac{n}{\sum _{i=1}^nx_i{k_i}^{\left( t\right) }} \end{aligned}$$

where $${k_i}^{\left( t\right) }=\delta (x_i;{\theta }^{\left( t\right) },{\beta }^{\left( t\right) }\ )$$ and $${r_i}^{\left( t\right) }=\left( 1-{\theta }^{\left( t\right) }\right) e^{-{\beta }^{\left( t\right) }x_i}$$.

### The information matrix 

We first calculate the elements of expected Hessian matrix of $\ell$ with respect to the distribution of *X*. According to that, let $a_{ij}$’s denote expected values of the second derivatives of $\ell$ with respect to $\theta ,\beta$ where $(i,j=1,2)$. Then we have

$$\begin{aligned} a_{11}&=E\left( \frac{{\partial }^2\ell }{\partial {\theta }^2}\right) \\&=\frac{-2n}{{\theta }^2}+\frac{4n}{{\left( 1+2\theta \right) }^2} -\,\frac{n{\theta }^2}{\left( 1+2\theta \right) {\left( 1-\theta \right) }^3}\left( \int _0^{1-\theta }\frac{r^2}{{\left( 3-r\right) \left( 1-r\right) }^3}dr-3\int _0^{1-\theta }\frac{r^2\left( 3-r\right) }{{\left( 1-r\right) }^5}dr\right)\\ &=\frac{-2n}{{\theta }^2}+\frac{4n}{{\left( 1+2\theta \right) }^2} -\,\frac{n{\theta }^2}{\left( 1+2\theta \right) {\left( 1-\theta \right) }^3}\left( \frac{9}{8}ln\left( \frac{2+\theta }{3\theta }\right) +\frac{1-5\theta }{4{\theta }^2}+1-\frac{3}{2}\frac{{\left( 1-\theta \right) }^3\left( 1+\theta \right) }{{\theta }^4}\right) \\ a_{22}&=E\left( \frac{{\partial }^2\ell }{\partial {\beta }^2}\right) =\frac{-n}{{\beta }^2}+\frac{12{n\theta }^2}{{\beta }^2\left( 1+2\theta \right) \left( 1-\theta \right) }\int _0^{1-\theta }\frac{r\left( 2-r\right) }{\left( 3-r\right) {\left( 1-r\right) }^5}{\left( \ln {\left( \frac{r}{1-\theta }\right) }\right) }^2dr\\ a_{12}&=a_{21}=E\left( \frac{{\partial }^2\ell }{\partial \theta \ \partial \beta }\right) =\frac{-12n{\theta }^2}{\beta \left( 1+2\theta \right) {\left( 1-\theta \right) }^2}\int _0^{1-\theta }\frac{r\left( 2-r\right) }{\left( 3-r\right) {\left( 1-r\right) }^5}\ln {\left( \frac{r}{1-\theta }\right) }dr \end{aligned}$$

Thus, Fisher information matrix, $\ I_n\left( \theta ,\beta \right)$ of sample size *n* for $\left( \theta ,\beta \ \right)$ is as follows:

$$\begin{aligned} I_n\left( \theta ,\beta \right) =- \begin{bmatrix} E\left( \frac{\partial ^2 \ell }{\partial {\theta }^2}\right)&\quad E\left( \frac{{\partial }^2 \ell }{\partial \theta \ \partial \beta }\right) \\ E\left( \frac{{\partial }^2 \ell }{\partial \theta \ \partial \beta }\right)&\quad E\left( \frac{{\partial }^2 \ell }{\partial {\beta }^2}\right) \end{bmatrix} = - \begin{bmatrix} a_{11}&\quad a_{12} \\ a_{12}&\quad a_{22} \end{bmatrix} \end{aligned}$$

Inverse of the Fisher-information matrix of single observation, i.e., $I_1^{-1}\left( \theta ,\beta \right)$ indicates asymptotic variance-covariance matrix of ML estimates of $\left( \theta ,\beta \right)$. Hence, joint distribution of maximum likelihood estimator for $\left( \theta ,\beta \right)$ is asymptotically normal with mean $\left( \theta ,\beta \right)$ and variance-covariance matrix $I_1^{-1}\left( \theta ,\beta \right)$. Namely,

$$\begin{aligned} \sqrt{n}\left( \left[ \begin{array}{ cc} \hat{\theta } \\ \hat{\beta } \end{array}\right] -\left[ \begin{array}{ cc} \theta \\ \beta \end{array}\right] \right) \sim AN\left( \left[ \begin{array}{ cc} 0 \\ 0 \end{array}\right] ,I_1^{-1}\left( \theta ,\beta \right) \right) . \end{aligned}$$

We have the 200 simulated data sets with sample size of $n=50$ from the EMDL distribution with known parameters as $\theta =0.6$ and $\beta =0.3$. Based on the asymptotic normal distribution, confidence ellipsoid of ML estimates for $\left( \theta ,\beta \right)$ can be drawn at the 95 % confidence level as follows.

Firstly, we present the asymptotic distribution of the ML estimates,

$$\begin{aligned} \sqrt{50}\left( \left[ \begin{array}{ cc} \hat{\theta } \\ \hat{\beta } \end{array}\right] -\left[ \begin{array}{ cc} 0.6 \\ 0.3 \end{array}\right] \right) \sim AN\left( \left[ \begin{array}{ cc} 0 \\ 0 \end{array}\right] ,{\left[ \begin{array}{ cc} 1.7622 & \quad -3.4018 \\ -3.4018 & \quad 10.3494 \end{array}\right] }^{-1}\right) \end{aligned}$$

**Fig. 7:**
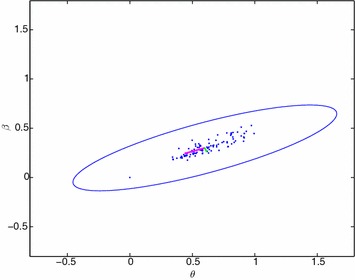
Confidence region for $\left( \hat{\theta },\hat{\beta }\right)$

Now, let $$\mu =\left[ \begin{array}{ cc} 0.6 \\ 0.3 \end{array}\right]$$ indicate the center of ellipsoid, and observed information matrix is calculated as $$\hat{I}\left( \theta ,\beta \ \right) =\left[ \begin{array}{ cc} 1.7622 & -3.4018 \\ -3.4018 & 10.3494 \end{array}\right]$$ (note that $\hat{I}\mathop {\rightarrow }\limits ^{P}I$). Then the confidence ellipsoid at the level 95 % is defined by $$50{\left( \left[ \begin{array}{ cc} \hat{\theta } \\ \hat{\beta } \end{array}\right] -\mu \right) }^{\prime}\hat{I}\left( \left[ \begin{array}{ cc} \hat{\theta } \\ \hat{\beta } \end{array}\right] -\mu \right) \le 5.99$$ where 5.99 is a critical value of the chi-squared distribution with two degrees of freedom with upper percentiles 95 % (Fig. 7).

### Simulation study

We conduct a simulation study generating 200 samples, each of which has a sample size of $n\ =10, 20, 50, 100$. We computed the moment (using lower and upper bounds) and ML (Newton-Raphson and EM algorithm) estimates of the parameters for every sample size level with different values of $\theta$ and $\beta$. From each generated sample of a given size *n* the root mean square errors (RMSE) of four estimates are also calculated. These results are tabulated in Table 1.

**Table 1:** Simulation results for moment, ML, EM estimates for different parameter values

Parameter $\left( \theta ;\beta \right)$	Sample size	Moment estimates (lower bound) $rmse\left( \hat{\theta };\hat{\beta }\right)$	Moment estimates (upper bound) $rmse\left( \hat{\theta };\hat{\beta }\right)$	ML estimates $rmse\left( \hat{\theta };\hat{\beta }\right)$	EM algorithm $rmse\left( \hat{\theta };\hat{\beta }\right)$
(0.01; 0.01)	10	(0.3819; 0.4290) (0.4305; 0.5161)	(0.5330; 0.7897) (0.6306; 0.9422)	(0.5560; 1.1057) (0.6350; 1.3591)	(0.4490; 0.7892) (0.5419; 1.0079)
	20	(0.3473; 0.4560) (0.4555; 0.5957)	(0.4871; 0.6023) (0.5500; 0.7010)	(0.3662; 0.5454) (0.4537; 0.6899)	(0.5202; 0.7779) (0.5953; 0.9440)
	50	(0.2734; 0.4128) (0.2933; 0.4678)	(0.1971; 0.2388) (0.2319; 0.3022)	(0.2927; 0.3971) (0.4197; 0.5767)	(0.2559; 0.3208) (0.2837; 0.3513)
	100	(0.2086; 0.2952) (0.2361; 0.3722)	(0.2050; 0.2653) (0.2250; 0.3083)	(0.1975; 0.2521) (0.2428; 0.3155)	(0.1806; 0.2556) (0.2135; 0.3216)
(0.01; 0.1)	10	(0.3441; 4.7886) (0.3940; 6.1487)	(0.4563; 6.0390) (0.4968; 7.6046)	(0.1563; 1.2578) (0.2539; 2.0050)	(0.5476; 8.4679) (0.6203; 10.1551)
	20	(0.4427; 6.1054) (0.5348; 8.0019)	(0.5069; 8.2839) (0.5310; 9.0880)	(0.1382; 1.4474) (0.3346; 3.2439)	(0.2243; 2.8182) (0.3571; 4.4648)
	50	(0.2810; 3.9128) (0.2982; 4.2335)	(0.3375; 4.8599) (0.3980; 6.1233)	(0.1457; 1.7248) (0.1635; 1.9789)	(0.3682; 5.9521) (0.4436; 7.3848)
	100	(0.2980; 4.2913) (0.3269; 4.9345)	(0.2281; 2.7206) (0.2440; 2.9904)	(0.2180; 3.1057) (0.2560; 3.6473)	(0.2187; 2.9054) (0.2819; 3.8716)
(0.01; 1)	10	(0.3455; 31.2778) (0.4174; 37.4113)	(0.3251; 31.8195) (0.3651; 40.4183)	(0.0320; 6.6267) (0.0714; 15.6975)	(0.1561; 21.8481) (0.1863; 24.7316)
	20	(0.4703; 66.2730) (0.5181; 78.6781)	(0.4256; 62.2380) (0.4959; 81.2181)	(0.0232; 1.8407) (0.0515; 3.9342)	(0.2677; 44.3097) (0.3238; 56.3634)
	50	(0.3570; 57.4291) (0.4121; 75.3072)	(0.3372; 45.2799) (0.4119; 63.8644)	(0.0485; 6.3230) (0.0690; 9.3401)	(0.2985; 42.1803) (0.3549; 53.6298)
	100	(0.2481; 35.1331) (0.2583; 37.8049)	(0.3825; 50.6405) (0.4070; 54.8860)	(0.0412; 5.3581) (0.0563; 8.0360)	(0.2263; 32.0492) (0.2549; 37.9289)
(0.01; 3)	10	(0.3955; 155.0823) (0.4273; 168.6059)	(0.3385; 118.2894) (0.3807; 151.7710)	(0.0169; 12.4376) (0.0274; 32.4726)	(0.2980; 215.2239) (0.3521; 305.7791)
	20	(0.4581; 185.6736) (0.5048; 211.9931)	(0.4414; 180.4513) (0.5424; 249.3666)	(0.0565; 22.2828) (0.0913; 38.1805)	(0.2077; 72.5677) (0.2928; 101.4907)
	50	(0.2804; 123.0569) (0.2960; 137.4734)	(0.3819; 129.9446) (0.4090; 145.2682)	(0.0301; 9.2323) (0.0470; 14.5592)	(0.1684; 68.5255) (0.2332; 96.9123)
	100	(0.2034; 78.9574) (0.2238; 90.6749)	(0.2601; 104.8366) (0.3032; 130.2829)	(0.0115; 3.6741) (0.0196; 6.4544)	(0.1746; 66.2565) (0.1975; 78.9489)
(0.1; 0.01)	10	(0.3717; 0.0426) (0.3629; 0.0535)	(0.5123; 0.0529) (0.4926; 0.0719	(0.4609; 0.0831) (0.4811; 0.1192)	(0.6697; 0.1255) (0.6698; 0.1715)
	20	(0.3217; 0.0443) (0.3398; 0.0530)	(0.4694; 0.0702) (0.4458; 0.0791	(0.4358; 0.0750) (0.4491; 0.0991)	(0.3200; 0.0492) (0.3301; 0.0603)
	50	(0.3540; 0.0474) (0.3450; 0.0524)	(0.2736; 0.0319) (0.2540; 0.0341)	(0.3502; 0.0459) (0.3586; 0.0492)	(0.1795; 0.0220) (0.1538; 0.0213)
	100	(0.1570; 0.0193) (0.1319; 0.0193)	(0.2562; 0.0264) (0.1824; 0.0204)	(0.1674; 0.0172) (0.1678; 0.0176)	(0.1996; 0.0220) (0.1450; 0.0159)
(0.1; 0.1)	10	(0.5543; 0.5408) (0.5215; 0.5529)	(0.5115; 0.6531) (0.4591; 0.7114)	(0.6810; 0.8712) (0.6672; 0.9190)	(0.5306; 0.6258) (0.5184; 0.7046)
	20	(0.4436; 0.5750) (0.4105; 0.5916)	(0.4167; 0.3775) (0.4206; 0.4234)	(0.4159; 0.4117) (0.4641; 0.4481)	(0.4431; 0.5409) (0.4599; 0.6300)
	50	(0.3695; 0.4175) (0.3499; 0.4394)	(0.2921; 0.2933) (0.2211; 0.2334)	(0.1822; 0.1846) (0.1566; 0.1769)	(0.2421; 0.3087) (0.1937; 0.2976)
	100	(0.1999; 0.2411) (0.1416; 0.2194)	(0.1993; 0.1916) (0.1396; 0.1535)	(0.2411; 0.2668) (0.2137; 0.2548)	(0.2038; 0.2215) (0.1343; 0.1502)
(0.1; 1)	10	(0.4461; 4.5711) (0.4257; 4.1085)	(0.5073; 5.6156) (0.4897; 7.4046)	(0.1549; 2.0957) (0.1488; 2.7522)	(0.6790; 7.3133) (0.6839; 7.5797)
	20	(0.3167; 3.8858) (0.3364; 5.1648)	(0.4691; 6.4103) (0.4813; 8.6758)	(0.1318; 1.4181) (0.1402; 1.7552)	(0.2355; 2.6073) (0.1795; 2.2731)
	50	(0.2459; 2.3518) (0.2212; 2.1697)	(0.2131; 2.1511) (0.1553; 1.7007)	(0.2238; 2.5573) (0.2083; 2.4655)	(0.2574; 2.8351) (0.1860; 2.2699)
	100	(0.1941; 2.2066) (0.1392; 1.7672)	(0.2142; 2.3646) (0.1853; 2.2036)	(0.1851; 2.0202) (0.1492; 1.8207)	(0.2388; 2.8016) (0.1965; 2.6684)
(0.1; 3)	10	(0.3444; 13.6255) (0.3562; 20.5479)	(0.4058; 11.4606) (0.3983; 11.9361)	(0.0083; 0.1631) (0.0950; 2.8788)	(0.2777; 8.0010) (0.3672; 10.6504)
	20	(0.3146; 7.9388) (0.3077; 7.7036)	(0.1761; 6.0556) (0.1258; 6.7467)	(0.0829; 2.6625) (0.0944; 2.9822)	(0.3651; 13.6145) (0.4316; 16.6203)
	50	(0.1978; 5.9274) (0.1392; 4.4230)	(0.2566; 8.6629) (0.2078; 8.3575)	(0.0943; 2.9838) (0.1105; 3.5267)	(0.2816; 9.8190) (0.2150; 8.2843)
	100	(0.1460; 4.0423) (0.0941; 2.5510)	(0.1929; 5.4612) (0.1277; 3.9084)	(0.1019; 3.3346) (0.0845; 3.2275)	(0.2194; 7.7674) (0.1791; 7.1549)
(0.6; 0.01)	10	(0.4846; 0.0113) (0.2573; 0.0063)	(0.7014; 0.0143) (0.2481; 0.0081)	(0.7000; 0.0109) (0.2921; 0.0052)	(0.7637; 0.0108) (0.3041; 0.0051)
	20	(0.4787; 0.0083) (0.2079; 0.0036)	(0.6509; 0.0109) (0.2240; 0.0061)	(0.5200; 0.0089) (0.2843; 0.0048)	(0.5983; 0.0111) (0.2889; 0.0064)
	50	(0.6111; 0.0106) (0.1988; 0.0033)	(0.6812; 0.0103) (0.2380; 0.0037)	(0.6782; 0.0115) (0.1719; 0.0026)	(0.7056; 0.0103) (0.1998; 0.0021)
	100	(0.5729; 0.0095) (0.1725; 0.0025)	(0.6413; 0.0099) (0.1502; 0.0021)	(0.6071; 0.0099) (0.1892; 0.0032)	(0.6108; 0.0100) (0.1777; 0.0027)
(0.6; 0.1)	10	(0.4892; 0.1172) (0.1959; 0.1301)	(0.6964; 0.1187) (0.1631; 0.0498)	(0.6865; 0.1116) (0.2722; 0.0345)	(0.6737; 0.1339) (0.2742; 0.0610)
	20	(0.6058; 0.1000) (0.2497; 0.0474)	(0.6651; 0.1040) (0.1942; 0.0334)	(0.5737; 0.0956) (0.3423; 0.0644)	(0.5784; 0.1081) (0.3023; 0.0697)
	50	(0.5760; 0.1028) (0.1671; 0.0279)	(0.6347; 0.1043) (0.1510; 0.0251)	(0.6598; 0.1162) (0.1668; 0.0321)	(0.6670; 0.1184) (0.2531; 0.0473)
	100	(0.6451; 0.0990) (0.1823; 0.0208)	(0.6198; 0.0950) (0.1734; 0.0270)	(0.6303; 0.1047) (0.1747; 0.0229)	(0.6188; 0.1014) (0.1253; 0.0206)
(0.6; 1)	10	(0.6795; 1.2128) (0.1585; 0.9660)	(0.6059; 1.0671) (0.1417; 0.5625)	(0.6832; 1.0181) (0.3404; 0.5152)	(0.6502; 1.5538) (0.3275; 1.1916)
	20	(0.6957; 1.2371) (0.2184; 0.5714)	(0.6458; 1.0511) (0.2042; 0.3870)	(0.5259; 0.8140) (0.2643; 0.4453)	(0.6212; 1.0367) (0.2875; 0.5246)
	50	(0.6684; 1.0214) (0.1378; 0.1628)	(0.6477; 1.0105) (0.1361; 0.3014)	(0.6550; 0.9959) (0.2374; 0.3766)	(0.6311; 0.9262) (0.2507; 0.3527)
	100	(0.6354; 1.0162) (0.1679; 0.2285)	(0.6323; 0.9949) (0.1456; 0.2346)	(0.6120; 1.0161) (0.1392; 0.2164)	(0.5899; 0.9988) (0.1893; 0.3216)
(0.6; 3)	10	(0.5661; 2.9674) (0.2097; 1.3517)	(0.5572; 3.1071) (0.2856; 2.7416)	(0.6525; 4.0879) (0.3489; 3.0165)	(0.5420; 2.8224) (0.2985; 1.7626)
	20	(0.6527; 3.6483) (0.2922; 1.9112)	(0.6443; 3.0669) (0.1563; 0.8436)	(0.6340; 3.2548) (0.2618; 1.4175)	(0.6871; 3.6108) (0.2115; 1.4077)
	50	(0.6667; 3.4335) (0.2030; 1.0722)	(0.5592; 2.7817) (0.2527; 1.4259)	(0.6313; 3.0814) (0.2326; 0.9826)	(0.6583; 3.1923) (0.1929; 1.1181)
	100	(0.6198; 3.0107) (0.1390; 0.6702)	(0.5831; 2.7708) (0.1301; 0.6725)	(0.6169; 3.0523) (0.1450; 0.6430)	(0.6374; 3.1514) (0.1758; 0.8689)
(0.9; 0.01)	10	(0.6143; 0.0071) (0.3933; 0.0048)	(0.7565; 0.0086) (0.2204; 0.0036)	(0.8192; 0.0107) (0.2439; 0.0033)	(0.7333; 0.0109) (0.3113; 0.0036)
	20	(0.6665; 0.0079) (0.3182; 0.0032)	(0.7730; 0.0083) (0.2121; 0.0032)	(0.7321; 0.0086) (0.2844; 0.0021)	(0.9318; 0.0099) (0.1212; 0.0025)
	50	(0.6855; 0.0085) (0.2732; 0.0024)	(0.8523; 0.0094) (0.1123; 0.0013)	(0.7905; 0.0096) (0.2251; 0.0026)	(0.9186; 0.0103) (0.1473; 0.0021)
	100	(0.7465; 0.0090) (0.2198; 0.0016)	(0.8935; 0.0103) (0.0622; 0.0011)	(0.8215; 0.0093) (0.1361; 0.0014)	(0.9268; 0.0100) (0.0812; 0.0009)
(0.9; 0.1)	10	(0.6462; 0.0862) (0.3381; 0.0378)	(0.7013; 0.0908) (0.2623; 0.0309)	(0.8431; 0.1020) (0.1934; 0.0374)	(0.9185; 0.1006) (0.2451; 0.0267)
	20	(0.8678; 0.1015) (0.1223; 0.0214)	(0.8388; 0.0995) (0.1228; 0.0269)	(0.7909; 0.1014) (0.2685; 0.0406)	(0.8270; 0.0988) (0.2316; 0.0419)
	50	(0.7725; 0.0956) (0.1698; 0.0121)	(0.7720; 0.0887) (0.2027; 0.0209)	(0.8624; 0.1042) (0.2035; 0.0226)	(0.7946; 0.0890) (0.2022; 0.0216)
	100	(0.7852; 0.0952) (0.1698; 0.0153)	(0.8895; 0.1031) (0.0870; 0.0180)	(0.9620; 0.1093) (0.1088; 0.0157)	(0.9496; 0.1016) (0.1028; 0.0099)
(0.9; 1)	10	(0.5889; 0.7639) (0.3829; 0.4564)	(0.6355; 0.9238) (0.3500; 0.4046)	(0.8911; 1.2604) (0.1467; 0.4797)	(0.8847; 1.0496) (0.1907; 0.3918)
	20	(0.6091; 0.7758) (0.3424; 0.4521)	(0.6895; 0.9191) (0.2611; 0.2828)	(0.7729; 0.8300) (0.2886; 0.3351)	(0.9399; 1.1832) (0.1065; 0.3144)
	50	(0.6475; 0.7636) (0.3288; 0.3296)	(0.8115; 0.9372) (0.2038; 0.1594)	(0.8992; 1.0015) (0.1707; 0.2070)	(0.8752; 0.9595) (0.1696; 0.1752)
	100	(0.7548; 0.8831) (0.2393; 0.2185)	(0.7883; 0.8887) (0.1605; 0.1674)	(0.9435; 1.1117) (0.1080; 0.1885)	(0.8962; 1.0487) (0.1629; 0.1792)
(0.9; 3)	10	(0.5325; 2.3452) (0.4575; 1.3839)	(0.8471; 3.0594) (0.1205; 0.6364)	(0.9126; 2.8102) (0.1976; 0.6489)	(0.8290; 2.9299) (0.2637; 1.3058)
	20	(0.6708; 2.3101) (0.3300; 0.9030)	(0.8174; 3.1643) (0.1580; 0.7843)	(0.8827; 3.0024) (0.1854; 0.7117)	(0.8202; 2.8625) (0.2537; 0.7691)
	50	(0.8508; 3.2611) (0.1162; 0.6055)	(0.8560; 2.8071) (0.1108; 0.4488)	(0.8717; 3.1389) (0.1911; 0.9120)	(0.9140; 2.9894) (0.1193; 0.3799)
	100	(0.8442; 2.7397) (0.1149; 0.4302)	(0.8867; 2.9404) (0.0769; 0.3865)	(0.8969; 2.9607) (0.1110; 0.3967)	(0.9193; 2.9727) (0.1070; 0.4539)

It is observed from the tables that when $\beta >\theta$, the ML estimates of $\theta$ and $\beta$ are better than the others with respect to the RMSE. When $\theta >\beta$, the moment estimates (both bounds) are as good as ML and EM estimates. Even for small sample size *n*, moment estimates are a little better.

## Applications

We illustrate the applicability of EMDL distribution by considering three different data sets which have been examined by a lot of other researchers. First data set is tried to be modeled by Transmuted Pareto and Lindley Distributions, second and third data sets are tried to be modeled by the Exponential-Poisson (EP) and Exponential-Geometric (EG) distributions. In order to compare distributional models, we consider some criteria as K-S (Kolmogorow-Smirnow), $-2LL$(−2LogL), AIC (Akaike information criterion) and BIC (Bayesian information criterion) for the data sets.

**Data Set1** The data consist of the exceedances of flood peaks (in m^3^/s) of the Wheaton River near Carcross in Yukon Territory, Canada. The data consist of 72 exceedances for the years 1958–1984, rounded to one decimal place. These data were analyzed by Choulakian and Stephens (2001) and are given in Table 2. Later on, Beta-Pareto distribution was applied to these data by Akinsete et al. (2008). Merovcia and Pukab (2014) made a comparison between Pareto and transmuted Pareto distribution. They showed that better model is the transmuted Pareto distribution (TP). Bourguignon et al. (2013) proposed Kumaraswamy (Kw) Pareto distribution (Kw-P). Tahir et al. (2014) have proposed weibull-Pareto distribution (WP) and made a comparison with Beta Exponentiated Pareto (BEP) distriubtion. Nasiru and Luguterah (2015) have proposed different type of weibull-pareto distribution (NWP). Mahmoudi (2011) concluded that the Beta-Generalized Pareto (BGP) distribution fits better to these data than the GP, BP, Weibull and Pareto models.

We fit data to EMDL distribution and get parameter estimates as $\hat{\theta }=0.7782$, $\hat{\beta }=0.0695$. According to the model selection criteria (AIC, or BIC) tabulated in Table 3, it is said that EMDL takes fifth place in amongst 10 proposed models.

**Table 2:** Exceedances of Wheaton river flood data

1.7	2.2	14.4	1.1	0.4	20.6	5.3	0.7
13.0	12.0	9.3	1.4	18.7	8.5	25.5	11.6
14.1	22.1	1.1	2.5	14.4	1.7	37.6	0.6
2.2	39.0	0.3	15.0	11.0	7.3	22.9	1.7
0.1	1.1	0.6	9.0	1.7	7.0	20.1	0.4
14.1	9.9	10.4	10.7	30.0	3.6	5.6	30.8
13.3	4.2	25.5	3.4	11.9	21.5	27.6	36.4
2.7	64.0	1.5	2.5	27.4	1.0	27.1	20.2
16.8	5.3	9.7	27.5	2.5	27.0	1.9	2.8

**Table 3:** Model selection criteria for river flood data

Model	K-S	−2LL	AIC	BIC
T. Pareto	0.389	572.401	578.4	580.9
Pareto	0.332	606.200	608.2	610.4
EP	0.199	574.600	578.6	583.2
BP	0.175	567.400	573.4	580.3
Kw-P	0.170	542.400	548.4	555.3
WP	–	498.793	502.8	507.3
NWP	–	158.326	162.3	166.9
BEP	–	496.111	504.1	513.2
BGP	0.071	486.200	496.2	507.6
EMDL	0.116	503.574	507.6	512.1

**Data Set2** The data set given in Table 4, contains the time intervals (in days) between coal mine accidents caused death of 10 or more men. Firstly, this data set was obtained by Maguire et al. (1952). There were lots of models on this data set such as Adamidis and Loukas (1998) and Kus (2007). They suggested to use Exponential-Geometric (EG) and Exponential-Poisson (EP) distributions respectively. On the other hand, Yilmaz et al. (2016) have proposed two-component mixed exponential distribution (2MED) for modeling this data set. In addition to these three models, we try to fit this data set by using EMDL distribution and we get the parameter estimates as $\hat{\theta }=\ 0.5239$ and $\hat{\beta }=\ 0.0025$. We have only K-S and p values which are tabulated in Table 5 to make a comparison.

According to Table 5, EMDL distribution fits better than EG distribution.

**Table 4:** The time intervals (in days) between coal mine accidents

378	96	59	108	54	275	498	228	217	19	156
36	124	61	188	217	78	49	271	120	329	47
15	50	1	233	113	17	131	208	275	330	129
31	120	13	28	32	1205	182	517	20	312	1630
215	203	189	22	23	644	255	1613	66	171	29
11	176	345	61	151	467	195	54	291	145	217
137	55	20	78	361	871	224	326	4	75	7
4	93	81	99	312	48	566	1312	369	364	18
15	59	286	326	354	123	390	348	338	37	1357
72	315	114	275	58	457	72	745	336	19

**Table 5:** K-S and p values for EP, EG, 2MED and EMDL

Model	K-S	p value
EP	0.0625	0.7876
EG	0.0761	0.5524
2MED	0.0578	0.8386
EMDL	0.0752	0.5436

**Data Set3** The data set in Table 6 obtained by Kus (2007) includes the time intervals (in days) of the successive earthquakes with magnitudes greater than or equal to 6 Mw. Kus (2007) has used this data set to show the applicability of the EP distribution and he made a comparison between EG and EP distributions with K-S statistic. Parameter esitmates of EMDL distribution are $\hat{\theta }=0.3540$, $\hat{\beta }=0.0003$. Calculated K-S statistic for EMDL can be seen in Table 7, according to this, EMDL distribution gives the best fit to earthquake data in three models.

**Table 6:** Time intervals of the successive earthquakes in North Anatolia fault zone

1163	3258	323	159	756	409
501	616	398	67	896	8592
2039	217	9	633	461	1821
4863	143	182	2117	3709	979

**Table 7:** K-S and p values for EP, EG and EMDL

Model	K-S	p value
EP	0.0972	0.9772
EG	0.1839	0.3914
EMDL	0.0706	0.9991

## Conclusions

In this paper we have proposed a new lifetime distribution, which is obtained by compounding the modified discrete Lindley distribution (MDL) and exponential distribution, referred to as the EMDL. Some statistical characteristics of the proposed distribution including explicit formulas for the probability density, cumulative distribution, survival, hazard and mean residual life functions, moments and quantiles have been provided. We have proposed bounds to solve moment equations. We have derived the maximum likelihood estimates and EM estimates of the parameters and their asymptotic variance-covariance matrix. Simulation studies have been performed for different parameter values and sample sizes to assess the finite sample behaviour of moments, ML and EM estimates. The usefulness of the new lifetime distribution has been demonstrated in three data sets. EMDL distribution fits better for the third data set consisting of the times between successive earthquakes in North Anatolia fault zone than the EP and EG.
